# Machine Learning Predicts Accurately *Mycobacterium tuberculosis* Drug Resistance From Whole Genome Sequencing Data

**DOI:** 10.3389/fgene.2019.00922

**Published:** 2019-09-26

**Authors:** Wouter Deelder, Sofia Christakoudi, Jody Phelan, Ernest Diez Benavente, Susana Campino, Ruth McNerney, Luigi Palla, Taane G. Clark

**Affiliations:** ^1^Faculties of Epidemiology & Population Health and Infectious & Tropical Diseases, London School of Hygiene & Tropical Medicine, London, United Kingdom; ^2^Dalberg Advisors, Geneva, Switzerland; ^3^Epidemiology and Biostatistics Department, Imperial College London, St Mary’s Campus, London, United Kingdom; ^4^Department of Medicine, University of Cape Town, Cape Town, South Africa

**Keywords:** *Mycobacterium tuberculosis*, MDR-TB, XDR-TB, drug resistance, machine learning

## Abstract

**Background:** Tuberculosis disease, caused by *Mycobacterium tuberculosis*, is a major public health problem. The emergence of *M. tuberculosis* strains resistant to existing treatments threatens to derail control efforts. Resistance is mainly conferred by mutations in genes coding for drug targets or converting enzymes, but our knowledge of these mutations is incomplete. Whole genome sequencing (WGS) is an increasingly common approach to rapidly characterize isolates and identify mutations predicting antimicrobial resistance and thereby providing a diagnostic tool to assist clinical decision making.

**Methods:** We applied machine learning approaches to 16,688 *M. tuberculosis* isolates that have undergone WGS and laboratory drug-susceptibility testing (DST) across 14 antituberculosis drugs, with 22.5% of samples being multidrug resistant and 2.1% being extensively drug resistant. We used non-parametric classification-tree and gradient-boosted-tree models to predict drug resistance and uncover any associated novel putative mutations. We fitted separate models for each drug, with and without “co-occurrent resistance” markers known to be causing resistance to drugs other than the one of interest. Predictive performance was measured using sensitivity, specificity, and the area under the receiver operating characteristic curve, assuming DST results as the gold standard.

**Results:** The predictive performance was highest for resistance to first-line drugs, amikacin, kanamycin, ciprofloxacin, moxifloxacin, and multidrug-resistant tuberculosis (area under the receiver operating characteristic curve above 96%), and lowest for third-line drugs such as D-cycloserine and Para-aminosalisylic acid (area under the curve below 85%). The inclusion of co-occurrent resistance markers led to improved performance for some drugs and superior results when compared to similar models in other large-scale studies, which had smaller sample sizes. Overall, the gradient-boosted-tree models performed better than the classification-tree models. The mutation-rank analysis detected no new single nucleotide polymorphisms linked to drug resistance. Discordance between DST and genotypically inferred resistance may be explained by DST errors, novel rare mutations, hetero-resistance, and nongenomic drivers such as efflux-pump upregulation.

**Conclusion:** Our work demonstrates the utility of machine learning as a flexible approach to drug resistance prediction that is able to accommodate a much larger number of predictors and to summarize their predictive ability, thus assisting clinical decision making and single nucleotide polymorphism detection in an era of increasing WGS data generation.

## Introduction

Tuberculosis (TB), caused by *Mycobacterium tuberculosis* bacteria, remains a major global public health challenge, with over 10.0 million people infected with TB and an estimated 1.6 million deaths in 2017 (World Health Organization, 2018a). An increasing prevalence of drug resistance presents a serious challenge to effective TB control (World Health Organisation, 2018b). First-line anti-TB therapy is centered around four drugs: rifampicin (RIF), isoniazid (INH), ethambutol (EMB), and pyrazinamide (PZA) (World Health Organization, 2017). *M. tuberculosis* strains resistant to at least RIF and INH are termed multidrug-resistant (MDR-TB), with >550,000 new resistant cases in 2017 (World Health Organisation, 2018b). Additional resistance to second-line drugs, the fluoroquinolones [FQ; ciprofloxacin (CIP), ofloxacin (OFL), or moxifloxacin (MOX)] and injectables [INJ; amikacin (AMK), kanamycin (KAN), capreomycin (CAP)], is termed extensively drug resistant (XDR-TB), and such cases have been reported in >115 countries (World Health Organisation, 2018b). Conventional TB treatment regimens are relatively long (>6 months) and include the simultaneous application of several drugs (World Health Organization, 2017). Treatment of drug-resistant TB is even more prolonged and involves drugs with severe side effects and with lower efficacy (World Health Organization, 2018a).

Anti-TB drugs act on *M. tuberculosis via* three main mechanisms: (i) blocking enzymes involved in the synthesis of components of the cell wall (e.g., EMB), (ii) disrupting protein synthesis at the level of the ribosomes [e.g., streptomycin (STM)] and (iii) hindering various processes at a DNA level such as RNA/DNA synthesis (e.g., RIF, FQ) (Nasiri et al., 2017). While *M. tuberculosis* drug-resistance mechanisms are not fully understood, they have been observed to be driven mainly by single nucleotide polymorphisms (SNPs) or other polymorphisms (e.g., small insertions and deletions, “indels”) resulting in the modification of drug targets (e.g., *rpoB* gene for RIF, *gidB* and *rpsL* genes for STM, *embB* gene for EMB, *gyrA* and *gyrB* genes for FQ, *rrs* gene for INJ) or in the loss of an ability to activate prodrugs (e.g., *katG* gene for INH, *pncA* gene for PZA) (Gygli et al., 2017). Mutations can be located within gene coding regions or within promoters [e.g., the *inhA* promoter for INH and ethionamide (ETH) resistance] (Palomino and Martin, 2014). A resistance mutation can directly alter drug action or be compensatory *via* activation of an alternative pathway. Mutations may cause resistance to multiple drugs and contribute to complex gene–gene interactions (Safi et al., 2013; Trauner et al., 2014; Gygli et al., 2017).

Drug resistance is traditionally diagnosed using bacterial culture and phenotypic testing, where uncovering resistance to first-line treatments leads to an assessment of second-line regimens. However, this approach is relatively slow and expensive, and it has inherent inaccuracies and reproducibility challenges (Farhat et al., 2016). Whole genome sequencing (WGS) is increasingly being used as a diagnostic tool to rapidly identify a wider set of mutations to inform clinical decision making (Dheda et al., 2017). WGS can also be used to identify new putative resistance loci, for example, through genome-wide association (GWAS) and phylogenetic-tree-based convergent evolution approaches (Coll et al., 2018). Classic regression methods, with and without the incorporation of regularization techniques, have been applied within a GWAS context to improve model generalizability and prevent model overfitting. However, these methods may fail to detect interactions among covariates and might be less suited to the analysis of large and high-dimensional datasets that arise from large-scale WGS projects (Lunetta et al., 2004; Hastie et al., 2009). This issue is of special relevance, as prior studies have indicated that there are likely to be as-yet undetected epistatic effects that might influence resistance (Farhat et al., 2016).

Machine learning is concerned with the development and application of computationally intensive analytical methods to extract information from complex datasets, with an emphasis on the task of prediction. With increasing numbers *of M. tuberculosis* clinical isolates undergoing WGS and the expanding numbers of loci implicated in resistance, machine learning offers a complementary approach to regression-based GWAS, as it has a superior capability to adapt to the growing body of clinical and biological data. Compared with regression, nonparametric machine learning methods such as classification trees (CTs) and gradient-boosted trees (GBTs) have few underlying model assumptions related to the distribution and functional relationships between the included covariates or predictors. They potentially provide greater flexibility for problems of prediction in high-dimensional variable spaces, when each individual covariate may contain limited information and covariate interactions are important (Lunetta et al., 2004; Heidema et al., 2007; Hastie et al., 2009). CTs and GBTs are recursive partitioning methods that have outperformed other classification techniques in genome-wide studies (Chen and Ishwaran, 2012) and provide predictions and the ranked importance of predictors as outputs (Efron and Hastie, 2017). GBTs in particular have achieved state-of-the-art results on many standard classification benchmarks and demonstrated scalability and speed, suggesting that they may perform well in drug-resistance studies (Chen and Guestrin, 2016). We aim to leverage the great interpretability of CTs with the superior prediction performance of GBTs.

Machine learning methods have previously been applied in a TB context, including to support digital X-ray analysis (Lakhani and Sundaram, 2017) and drug development and to assess antitubercular properties of compounds (Periwal et al., 2011). In the context of predicting pathogen drug resistance, researchers have looked to apply random forest classification and GBT models (Farhat et al., 2016; Yang et al., 2018; Kouchaki et al., 2018). For TB, different statistical models have been applied to different drugs within the same study, rather than adopting a single approach across all drugs (Kouchaki et al., 2018). Our approach differs from these and other studies in one or more of the following aspects. First, our dataset is one of the largest for TB, consisting of nearly 17,000 *M. tuberculosis* isolates sourced globally, and considers phenotypic data for a wider range of drugs (*n* = 14), including for less often used ones such as para-aminosalisylic acid (PAS), cycloserine (CYS), and ETH. Not only do we focus on known drug-resistance SNPs or genes, but we also analyze (640K) genome-wide SNPs with an opportunity to inform new variant discovery. Therefore, our dataset provides a unique opportunity to evaluate machine learning methods, which could be rolled in a clinical setting, based on actual *M. tuberculosis* “big data.” Second, we use a combination of CTs and GBTs to optimize resistance prediction and SNP discovery (Hastie et al., 2009). Third, we assess the impact and implications of including “co-occurrent resistance” markers in the prediction models. These are mutations that are known to be causing resistance to other drugs. Furthermore, we have developed a new approach to graphically interpret and rank the results of the GBT models and propose approximate novel SNP detection thresholds, supporting the detection and interpretation of putative new SNPs linked to drug resistance. In summary, we investigate the potential of applying cutting-edge CT and GBT machine learning methods to predict drug resistance and thereby support surveillance and clinical decision making, as well as assist the discovery of putative new SNPs linked to resistance.

## Results

### *M. Tuberculosis* Sequence Data, Genetic Diversity, and Drug Resistance

**Figure 1:**
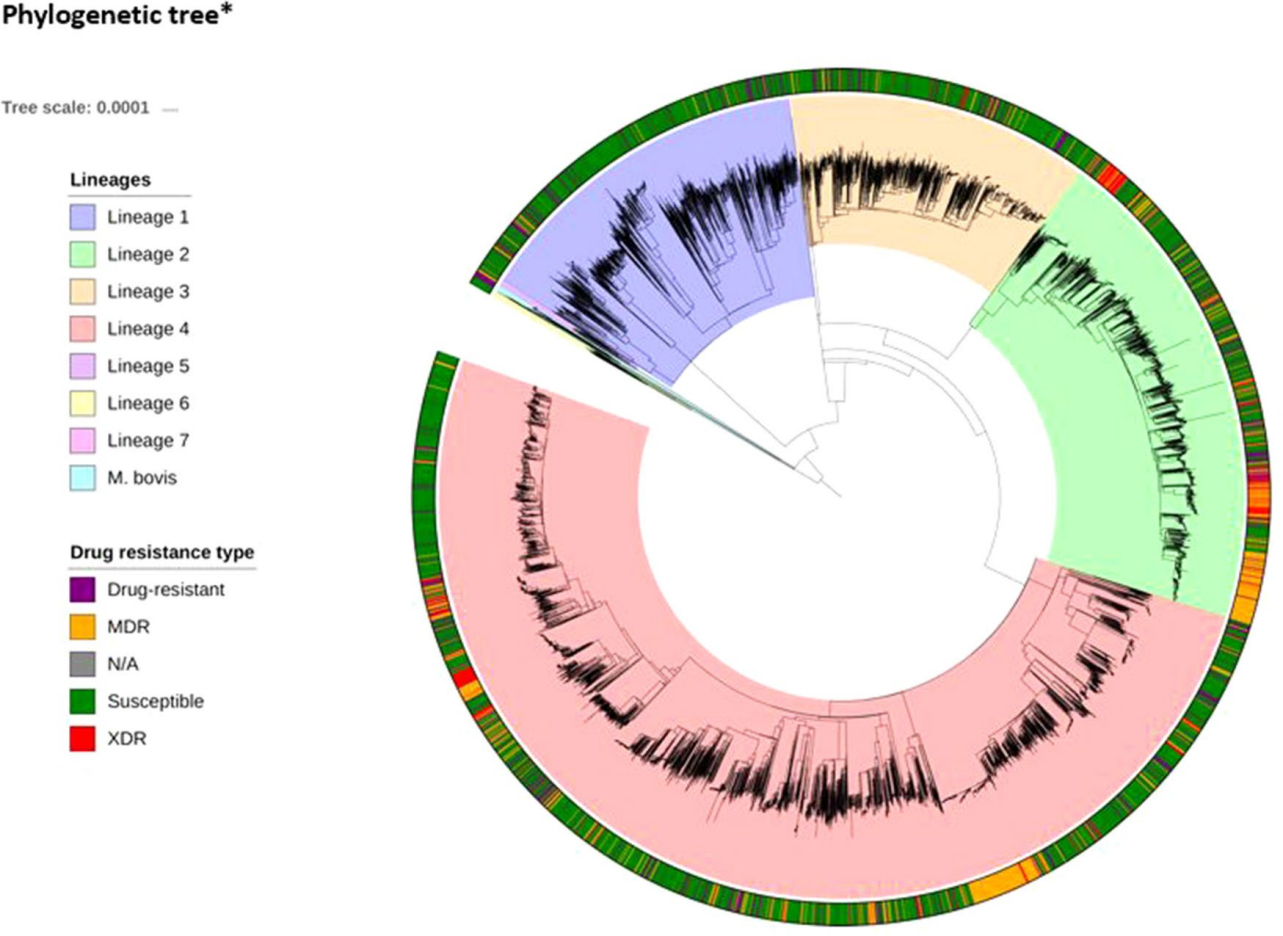
Phylogenetic tree*(attached as separate file)* The tree includes all 16,688 isolates, complemented by additional data from lineages 5–7 and *M. bovis*. The tree was fitted using a maximum likelihood approach implemented in RAxML (Stamakis, 2014).

WGS and drug susceptibility testing data were available across 16,688 isolates (S1 Table), which cover the four main lineages (L1, 11.1%; L2, 21.9%; L3, 17.0%; L4, 50.1%; S2 Table). Across the isolates, 642,580 high-quality genome-wide SNPs were identified, with the majority in genic regions (91.6%; 56.9% of mutations leading to nonsynonymous amino acid changes). The majority of SNPs (98.9%) have low minor allele frequencies (< 1%). We also included covariates representing the aggregation of nonsynonymous mutations by locus within our machine learning approach. A phylogenetic tree constructed using all genome-wide SNPs revealed the expected clustering by lineage (**Figure 1**). The CT and GBT approaches implemented also selected lineage-specific markers to account for the phylogeographic-based population stratification.

Laboratory drug susceptibility testing (DST) of anti-TB drugs found that 35.5% of isolates had a resistance phenotype (MDR-TB, 22.5%; XDR-TB, 2.1%; other, 11.0%; **Table 1**; **S2 Table**; **S3 Table**). Due to oversampling, these rates are higher than those typically seen in clinical or surveillance settings. Fourteen drugs were included in the genome-wide analysis: INH, RIF, ETH, PZA, EMB, STM, AMK, CAP, KAN, CIP, OFL, MOX, CYS, and PAS, as well as the composite MDR-TB phenotype. Phenotypic DST data were not available for every isolate across each of the 14 drugs, as only those individuals resistant to first-line treatments are typically tested for second-line resistance. Therefore, the number of samples tested ranged from >16,000 for the most commonly tested first-line drugs (INH and RIF; ≥98.0%) to <407 (≤2.4%) for less often phenotypically assessed drugs such as PAS, CYS, and CIP (**S3 Table**). Insufficient phenotypic data were available for the inclusion of the new and repurposed drugs such as bedaquiline, delamanid, and linezolid as well as for XDR-TB.

**Table 1:** Drug-resistance loci identified in the machine learning models.

Drug	*N*	Resistant	%	CT-KDG (N)	CT-ALL (N)	GBT-ALL (N)	Overlapping Loci
Isoniazid	16,422	5,215	31.8	2	5	103	katG*, fabG
Rifampicin	16,507	4,462	27.0	1	1	39	rpoB*
Pyrazinamide	11,968	1,813	15.1	2	4	116	*pncA*
Ethambutol	14,830	2,576	17.4	1	10	36	embB*
Streptomycin	5,213	1,338	25.7	4	4	134	rpsL*, rpsl, rrs*, rrs
Amikacin	1,435	335	23.3	1	1	35	*rrs*
Capreomycin	1,731	389	22.5	1	3	44	*rrs*
Kanamycin	1,843	639	34.7	1	2	43	*rrs*
Ciprofloxacin	400	63	15.8	1	1	30	gyrA*
Ofloxacin	1,993	506	25.4	1	1	42	gyrA*
Moxifloxacin	885	104	11.8	1	2	36	gyrA*
Ethionamide	940	329	35.0	3	1	60	fabG*
Cycloserine	391	105	26.9	1	5	44	*alr*
PAS	407	43	10.6	1	1	54	*folC*
MDR-TB	–	3748	22.5	1	1	82	rpoB*, katG, fabG

PAS, para-aminosalisylic acid; CT-KDG is a classification tree (CT) applied to a dataset with SNPs that are known to be associated with drug resistance [derived from Ref. (Phelan et al., 2019)]; CT-ALL and GBT-ALL are, respectively, a CT and gradient boosted tree (GBT) applied to a dataset that includes all genome-wide SNPs, except those linked to resistance for other drugs (co-occurrent resistance markers); GBT-CRM is a GBT that is applied to all genome-wide SNPs; MDR-TB is multidrug resistant TB, that is, resistance to isoniazid and rifampicin. *Total number of nonsynonymous mutations in that gene.

### Machine Learning Models to Predict Drug Resistance

CT and GBT approaches were used to predict drug resistance and support new SNP discovery. We fitted CT models using datasets either consisting of SNPs in genes known to be linked to drug resistance (CT-KDG) or genome wide (CT-ALL). One GBT model was fitted to datasets with all genome-wide SNPs (GBT-ALL). All of these three models (CT-KDG, CT-ALL, and GBT-ALL) excluded known co-occurrent resistance markers. We fitted one additional approach (GBT-CRM) that included all genome-wide SNPs and, therefore, potential co-occurrent resistance markers in the model. Finally, for the purpose of comparison, we fitted a logistic regression (LR) model on the SNPs in genes known to be linked to drug resistance (LR-KDG). For all approaches, we also included the aggregated count of all nonsynonymous mutations per gene in the dataset, to allow the models to use this covariate as a potential starting point and potentially cover known resistance mutations that have low frequency (Phelan et al., 2019). It should be noted that the dataset did not contain large deletions, which we have found to be present in some resistant isolates, but at very low frequency overall (Coll et al., 2018). The resulting CT-KDG models included between one and four SNPs or loci. For the CT-ALL and GBT-ALL, the number of predictors selected varied from 1 to 10 and from 30 to 134, respectively (**Table 1**), and included lineage or strain-specific markers that are not causally linked to resistance. All models overlapped with respect to known drug-resistance loci (**Table 1**), confirming that they are the strongest predictors of resistance. In some cases, the CT-KDG and CT-ALL models were identical (e.g. RIF, EMB, AMK, CAP, CIP, OFL).

### The Performance of the Machine Learning Models

The predictive performance of the machine learning approaches was assessed by calculating the sensitivity and specificity and the area under the receiver operating characteristic curve (AUC), assuming the laboratory DST result was the gold standard (**Table 2**). The GBT-CRM sensitivity for RIF (88.8%) and INH (91.1%) was higher than for EMB (82.8%) and PZA (69.7%). The sensitivity for fluoroquinolones was highest for CIP (85.7%), followed by OFL (81.0%) and MOX (53.3%). The sensitivity for the injectables was highest for KAN (82.2%), followed by AMK (80.5%) and CAP (74.6%). The model sensitivity for the remaining drugs [ETH (68.1%), CYS (50.0%), and PAS (20.0%)] is substantially lower. The overall sensitivity for MDR-TB was 90.4%. The GBT-ALL model tended to outperform the CT models, with respect to sensitivity and specificity, and CT-ALL had stronger performance than CT-KDG. The AUC values for most major first- and second-line drugs for the GBT model were above 90% (and often above 95%) (**S4 Table**). The overall predictive performance across models for CYS and PAS was relatively weak. In general, larger datasets with well-characterized PAS and CYS phenotypes will be needed to assist with identifying the full repertoire of related resistance mutations (Farhat et al., 2016; Coll et al., 2018).

**Table 2:** Sensitivity, specificity, and accuracy for the models (maximum value per prediction measure is bolded).

Drug	LR-KDG			CT-KDG			CT-ALL			GBT-ALL			GBT-CRM		
	Sens.	Spec	Acc.	Sens	Spec	Acc	Sens	Spec	Acc	Sens	Spec	Acc	Sens	Spec	Acc
INH	87.3	**99.1**	95.3	87.3	**99.1**	95.3	87.3	**99.1**	95.3	88.0	99.0	95.4	**91.1**	98.8	**96.3**
RIF	82.8	**99.6**	95.1	82.8	**99.6**	95.1	82.8	**99.6**	95.1	82.8	**99.6**	95.1	**88.8**	98.9	**96.2**
PZA	21.6	**100**	87.2	21.6	**100**	87.2	35.2	98.5	88.2	42.8	99.2	90.0	**69.7**	96.1	**91.8**
EMB	**84.7**	93.1	91.6	80.9	94	91.6	80.9	94.0	91.6	81.7	**94.7**	**92.4**	82.8	94.2	92.1
STM	71.6	**97.8**	91.1	72.3	96.5	90.3	71.2	97.3	90.6	72.3	97.3	90.9	**79.8**	96.0	**91.9**
AMK	**80.5**	**99.5**	**95.1**	**80.5**	**99.5**	**95.1**	**80.5**	**99.5**	**95.1**	**80.5**	**99.5**	**95.1**	**80.5**	**99.5**	**95.1**
CAP	69.6	95.5	89.6	69.6	95.5	89.6	69.6	95.5	89.6	72.1	95.8	90.4	**74.6**	**96.2**	**91.3**
KAN	74.4	**99.1**	89.7	74.4	**99.1**	89.7	**82.2**	97.8	91.8	80.8	97.8	91.3	**82.2**	98.2	**92.1**
CIP	**92.8**	**98.5**	**97.5**	**92.8**	**98.5**	**97.5**	**92.8**	**98.5**	**97.5**	85.7	**98.5**	96.2	85.7	**98.5**	96.2
OFL	80	**97.7**	93.5	80.0	**97.7**	93.5	80.0	**97.7**	93.5	**81.0**	**97.7**	**93.7**	**81.0**	97.0	93.2
MOX	**66.6**	93.2	90.9	**66.6**	93.2	90.9	46.6	**98.1**	**93.7**	53.3	96.2	92.6	53.3	97.5	**93.7**
ETH	**75.7**	75.6	75.6	**75.7**	75.6	75.6	74.2	79.6	77.7	66.6	92.6	83.5	68.1	**93.4**	**84.6**
CYS*	**57.6**	88.6	**78.4**	38.4	**98.1**	**78.4**	30.7	94.3	73.4	46.1	92.4	77.2	50.0	92.4	**78.4**
PAS	0	**100**	87.8	**20.0**	**100**	**90.2**	0	**100**	87.8	10.0	**100**	89.0	**20.0**	**100**	**90.2**
MDR	85.9	96.9	94.4	85.9	96.9	94.4	85.9	96.9	94.4	86.2	**97.5**	95.0	**90.4**	96.9	**95.5**

*No known drug-resistance SNPs for CYS were included in the KDG models; reported outcomes are the performance on the test set; RIF, rifampicin; INH, isoniazid; EMB, ethambutol; PZA, pyrazinamide; CIP, ciprofloxacin; OFL, ofloxacin; MOX, moxifloxacin; AMK, amikacin; KAN, kanamycin; CAP, capreomycin; PAS, para-aminosalisylic acid (PAS); CYS, cycloserine; ETH, ethionamide; CT-KDG is a classification tree (CT) fitted to a dataset with SNPs that are known to be associated with drug resistance [derived from Ref. (Phelan et al., 2019)]; LR-KDG is a logistic regression model applied to the same SNP set as CT-KDG; CT-ALL and GBT-ALL are, respectively, a CT and gradient boosted tree (GBT) applied to a dataset that includes all genome-wide SNPs, except those linked to resistance for other drugs (co-occurrent resistance markers); GBT-CRM is a GBT that is applied to all genome-wide SNPs; MDR is multidrug resistant TB.

#### Comparison Between GBT-CRM and Other Machine Learning Models

Owing to the inclusion of co-occurrent resistance markers, the GBT-CRM model was almost always the best in terms of predictive accuracy and AUC, with a marked improvement for PZA and PAS (**S1 Table**). The GBT-ALL model, which excludes co-occurrent resistance markers, but can include marker interactions and strain markers, also tended to outperform the KDG models, but to a lesser extent than GBT-CRM. The difference in predictive performance between the GBT-ALL and the KDG models was especially large for ETH and CYS.

#### Comparison With an in *Silico* Panel of Known Mutations and GWAS

We also compared the predictive abilities of GBT-ALL, CT-ALL, and CT-KDG models to those from the TB-Profiler mutation panel consisting of >1,300 markers across the 14 drugs (**S5 Table**) (Coll et al., 2015; Phelan et al., 2019). First, we used only those markers with minor allele frequency of >0.5% to predict resistance (“TB Panel”; **S6 Table**) and attained a performance similar to KDG models (**Table 2**). We then used the TB-Profiler (full) mutation panel and software (Phelan et al., 2019), which rules in observed frameshift mutations, large deletions, and missense mutations in known resistance genes. As TB-Profiler includes mutations occurring at low frequencies, the predicted accuracy was superior than the machine learning approaches for most drugs. For five drugs, where the resistance mechanisms are less understood, including STM, ETH, and PAS, the GBT-CRM model had a marginally better performance than the TB-Profiler (**S6 Table**). We also compared the predictive abilities of the GBT-CRM to those from an updated GWAS analysis [similar implementation to (Coll et al., 2018)] (**S6 Table**). Overall, the accuracy of both models was in the same range (<1% difference) for most drugs, with the exception for CAP, KAN, and CYS, where the performance of GWAS was distinctively greater, and with exception for PZA, MOX, and ETH, where the performance of GBT-CRM was better.

#### Comparison With Other Studies That Apply Machine Learning Methods

We compared our models to the results of four recent studies that have applied different machine learning models (Yang et al., 2018; Kouchaki et al., 2018; Chen et al., 2019; Yang et al., 2019). Specifically, we compared both the average and maximum of the reported results for each metric (sensitivity, specificity, AUC) for each drug across the four studies (**S7 Table**; **S8 Table**). All the comparator studies included co-occurrent resistance markers. The specificities tended to be greater for the GBT-CRM model. The sensitivities tended to be greater for one or more of the models used in the other studies. However, overall, for six drugs (PZA, AMK, CAP, KAN, CIP, and MOX), the AUC scores of the GBT-CRM were higher than for the best model for that specific drug in other studies.

### Detection and Interpretation of Putative New SNPs

The CT-ALL and GBT-based approaches did not discover any putative new SNPs that met the stringent detection thresholds. We present and display a new visual approach to mutation ranking that leverages the output of the GBT-ALL model (**S1 Fig**). A number of known candidates (e.g., Rv1463 for RIF resistance) presented with marginal evidence.

## Discussion

With the rollout of WGS-based TB diagnosis across many countries (including UK) (PHE, 2018), there is a need to develop global TB datasets and databases (Coll et al., 2018; ReSeqTB, 2018), which in turn will require the implementation of “big data” analytical approaches (e.g., machine learning methods) to assist clinical and control program decision making. We have shown that CT and GBT machine learning approaches can play a value-adding role in predicting drug resistance and the possible detection of new putative variants. In general, the predictive performance of the CT models was inferior to the GBT approaches, but they captured the most common mutations driving resistance. When using aggregated counts of nonsynonymous mutations in known resistance genes as a predictor in the trees, the CT models did not include any known individual SNPs in that respective gene in an exclusionary manner as an additional predictor. This observation provides not only support for the validity and accuracy of the overall TB-Profiler lists but also the use of aggregation as a first parse approach to identifying relevant genes. The possible exception relates to KAN, CAP, and AMK, where the machine learning models chose a subset of the list of TB-Profiler SNPs.

The predictive performance of the GBT models, and especially the GBT-CRM model, is similar or higher than that of the models developed in other studies (Yang et al., 2018; Kouchaki et al., 2018; Chen et al., 2019; Yang et al., 2019). The performance of the more complex GBT models (GBT-ALL and GBT-CRM) in some cases is worse than TB-profiler (Phelan et al., 2019), but the comparison is affected by the fact that the latter approach uses rare alleles and deletions for prediction. For some drugs where the resistance mutations are not fully established (e.g., CYS, STM, and PAS), the GBT-CRM model had a similar or better predictive performance to the TB-profiler panel. The improved performance of the GBT-CRM over GBT-ALL and CT models may be explained by its ability to capture covariate interactions and the inclusion of co-occurrent resistance markers and strain-specific SNPs that may be informative in resistance outbreaks but in themselves may be related to transmissibility and not drug resistance. The inclusion of co-occurrent resistance markers might lead to overoptimism in the estimated performance that may not translate optimally into clinical practice. This optimism bias affects both prediction as well as detection (i.e., through mutation ranking) and may be caused by an interplay between high DST measurement errors (e.g., for pyrazinamide) (APHL, 2016), sequential testing, data from settings where drug availability is unregulated, the structure and stratification of the datasets, and differential resistance mechanisms not captured in a database (e.g., Lisboa strain types which have different MDR-TB mutations) (Coll et al., 2018). Ideally, resistance predictions should be based on underlying biological mechanisms, with co-occurring mutations having little effect, thereby assisting with the identification of novel putative markers and pathways. While our machine learning analysis suggested no novel SNPs at the importance thresholds used, in general, the approach ranks the informativeness of SNP mutations, which assists the detection of novel polymorphisms. As databases get larger with greater numbers of well-characterized resistance samples, especially for third-line drugs, there is improved potential to identify novel resistance mutations using machine learning approaches.

As expected, the overall predictive ability of INH, RIF, and MDR-TB resistance across the machine learning approaches was high (∼90% sensitivity) because the underlying mutations and loci involved are well established. However, 10% of resistance cases were not identified by the models. The genotypic–phenotypic discordance, as measured with the GBT-ALL model, was higher for other first-line (e.g., EMB, ∼20% and PZA, ∼60%) and second-line drugs (AMK and CAP, ∼20–25%; ETH, ∼35%; CYS, ∼55%), and large discrepancies point towards unknown genetic factors. However, other factors potentially have an effect, including laboratory DST errors or misspecified or truncated drug assay breakpoints (World Health Organization, 2018c), efflux-pump upregulation (Balganesh et al., 2012; Gygli et al., 2017), and epigenetic and hetero-resistance effects (Folkvardsen et al., 2013; Farhat et al., 2016). For example, the recent downward revision of the critical concentrations for the fluoroquinolones and injectables is likely to decrease specificity and increase sensitivity of WGS-based analysis (World Health Organization, 2018c). Future studies should aim to use quantitative minimum inhibitory concentration scores as phenotypes (Farhat et al., 2018). For heteroresistance, both resistance and wild-type mutations occur in a mixed infection. If the resistant strain has a relatively low abundance, the drug may be labeled resistant according to the DST result but sensitive in genomic sequencing (Folkvardsen et al., 2013; Farhat et al., 2016), leading to false negative results. Across the 32 drug targets in the TB-Profiler mutation library, 28 appear to have some evidence of heteroresistance within the 17k dataset (Phelan et al., 2019). With the lower error rates and higher depth of WGS, the detection of such low frequency variants is possible; therefore, combined with robust bioinformatic approaches, sequencing is being viewed as the gold standard for drug resistance characterization (Coll et al., 2018).

In summary, our approach has shown that machine learning can robustly predict drug resistance and inform on its underlying mutations. Furthermore, such approaches will be scalable when WGS becomes routine and increasingly “big data” analyses are required.

## Materials and Methods

### Phenotypic and Sequencing Data

The dataset consists of 16,688 isolates (lineages 1–4) with WGS data and phenotypic DST data (see **S1 Table** for accession numbers). The laboratory drug susceptibility testing followed WHO recommended protocols and practice [see Ref. (Coll et al., 2018)]. The raw sequence data were mapped to the H3Rv reference genome using *bwa-mem* software, and SNPs and insertions and deletions (indels) called from the consensus of GATK and *samtools* software. The final set of SNPs (*N* = 642,580) and indels included those with low levels of missing genotypes (<2%) and excluded those in the hypervariable PE/PPE gene families. Missing values were imputed using a nearest neighbor imputation approach. The dataset was augmented with covariates that aggregated the number of nonsynonymous mutations isolated in a locus.

### Fitting the Machine Learning Models

CTs (Hastie et al., 2009) were created from two SNP sets: one based on those in known drug resistance genes (Coll et al., 2015) (*N* = 1,421 SNPs; “CT-KDG”) and the other using all SNPs in the dataset (*N* = 641,159, “CT-ALL”). CT algorithms produce only one easy to interpret tree as output. GBT models (Friedman, 2000; Hastie et al., 2009) were fitted to a genome-wide SNP dataset (GBT-ALL), leading to an ensemble of short and stumpy decision trees constructed in an adaptive manner. The GBT models allowed us to move beyond binary inclusion of SNPs in the final model and assess, for the purpose of SNP discovery, the weight and importance of the SNPs included. The LR model was applied to the same set of SNPs as the CT-KDG model. As mentioned, we excluded known resistance markers for drugs that were not the phenotype of interest in each individual model in the logistic regression LR-KDG, CT-KDG, CT-ALL, and GBT-ALL, but included these markers in the GBT-CRM approach.

We created a split in the dataset where 80% was used as a training and validation set, and 20% was used as a test set. We applied five-fold cross-validation to the training set to calculate the prediction accuracy and used this to select the maximum depth parameter of the CT and GBT models. (Hastie et al., 2009). The penalized LR model was cross-validated on the regularization strength C for the L1 penalty. The final models were trained on the training set and were subsequently applied to the test set, with those outcomes reported in the Results section. For the CT models, the maximum depth parameter was selected as the smallest value that was within one standard error from the best performing maximum depth setting. We followed this “one-standard-error” rule to further induce the selection of parsimonious models and to mitigate the risk of over-fitting (Hastie et al., 2009). In both the GBT and CT models, the predictions in the final leaf nodes of the tree were determined by the majority class in those nodes. The reported scores (sensitivity, specificity, accuracy, positive predicted value, negative predicted value, and AUC) were calculated after fitting the model to the training dataset with the maximum depth as described per above and other parameter values (described in **S9 Table**). The GBT models are based on an ensemble of 50 trees (to facilitate a consistent comparison across drugs with regards to the mutation ranking) with a subsampling of 60% of isolates to fit each tree. These models provide a score for weight, coverage, and importance. The “weight” refers to the number of times a feature (covariate) appears in a tree/forest; “coverage” is the relative quantity of observations affected by a feature (which would be higher for covariates that are higher up in the tree), and “importance” is the average gain in the predictive accuracy when a SNP is chosen to split a tree node. SNP discovery using GBTs was assisted by construction of a two-dimensional mutation-ranking graph (see **S1 Figure**) displaying importance gain versus weight, with coverage as the bubble size. Those SNPs with high importance and weight are more likely to be predictive in a large number of trees across different subsamples of the data and, therefore, more generalizable. The suggested thresholds for the importance and weight were chosen pragmatically based on the inclusion of known and established resistance markers. These thresholds are shown as dotted lines on the graphs (**S1 Figure**).

The core packages used in the analysis included the *SHAP* (Lundberg and Lee, 2017) to visualize the relative contribution of each predictor, the decision tree classifier in *sklearn* (version 0.19.1), and the *Xgboost* implementation (version 0.70) was used to construct the CTs and GBTs (Chen and Guestrin, 2016). The default settings were used for the implementation of these machine learning algorithms, with the exception of the parameters as specified (see **S9 Table**). The plausibility of putatively causal SNPs identified was assessed through a search of the literature, including for gene function on *Mycobrowser* (Kapopoulou et al., 2011).

### Comparisons to Mutation Libraries, GWAS, and Other Studies.

We compared our machine learning prediction results to those from using a set of known SNPs associated with drug resistance on a rule-in basis. A first comparison was made with predictions based on mutations in the TB-Profiler panel (Phelan et al., 2019) that were common (minor allele frequency > 0.5%) in our dataset (TB-Panel). A second comparison was made with the application of the TB-Profiler software and its full mutation library (Phelan et al., 2019) to the dataset. We also compared our results to the application of a mixed-model regression GWAS approach (Coll et al., 2018) to the ∼17k dataset, as well as other studies that applied machine learning methods (Yang et al., 2018; Kouchaki et al., 2018; Chen et al., 2019; Yang et al., 2019).

## Data Availability Statement

The raw whole genome sequencing data is available from the European Nucleotide Archive (ENA) (**S1 Table**). The computing code is available upon request from the corresponding authors.

## Author Contributions

WD, SCa, RM, LP, and TC conceived and designed the study. JP and EB performed the bioinformatic processing of the raw sequencing data and phenotypic data. WD performed the statistical analysis, under the supervision of LP and TC. SCa performed a statistical analysis on a subset of the data, under the supervision of RM and TC. WD wrote the first draft of the manuscript, and the final version included edits from all authors. The final manuscript was read and approved by all authors.

## Funding

JP is supported by a Newton Institutional Links Grant (British Council) (261868591). SCa is funded by Medical Research Council UK grants (MR/M01360X/1, MR/R025576/1, and MR/R020973/1). TC is funded by the Medical Research Council UK (Grant no. MR/M01360X/1, MR/N010469/1, MR/R025576/1, and MR/R020973/1) and BBSRC (Grant no. BB/R013063/1).

## Conflict of Interest

Author WD was employed by the company Dalberg Advisors in Switzerland. The remaining authors declare that the research was conducted in the absence of any commercial or financial relationships that could be construed as a potential conflict of interest.
