# Supplementary material for: Machine Learning Predicts Accurately Mycobacterium tuberculosis Drug Resistance From Whole Genome Sequencing Data
Source: Front Genet. 2019 Sep 26;10:922. doi: 10.3389/fgene.2019.00922 (PMC6775242; doi:10.3389/fgene.2019.00922)
Supplement: Supplementary file 1 [file DataSheet_1.pdf]

S1 Table

Sources of sequence data, and drug resistance phenotypes

Project	N	Susceptible	Drug-resistant	MDR-TB	XDR-TB
Mixed	8128	5005	744	2379	0
PRJNA282721	1840	1471	277	87	5
PRJEB2794	1306	1219	79	8	0
PRJEB7056	1088	874	173	41	0
PRJEB9680	1031	710	73	246	2
PRJEB10385	682	98	193	296	95
PRJEB2221	356	331	19	6	0
PRJNA183624	331	85	41	138	67
PRJEB2358	325	293	30	2	0
PRJEB7669	232	0	3	218	11
PRJNA235852	208	155	33	20	0
PRJEB5162	191	175	14	2	0
PRJNA187550	157	43	0	91	23
PRJEB11653	126	14	77	35	0
PRJNA200335	126	23	5	43	55
PRJEB14199	123	0	34	14	75
PRJEB2777	98	98	0	0	0
PRJEB7281	95	38	15	41	1
PRJEB6945	46	46	0	0	0
PRJEB2424	45	3	2	40	0
PRJEB15857	38	18	5	15	0
PRJEB2138	37	9	4	14	10
PRJNA49659	30	30	0	0	0
PRJEB7727	28	12	11	5	0
PRJNA376471	18	11	0	7	0
PRJEB6276	3	3	0	0	0
Total	*16688*	*10764*	*1832*	*3748*	*344*

* https://www.ebi.ac.uk/ena; Drug-resistant refers to non-MDR-TB/-XDR-TB resistance; MDR-TB is defined as resistance to isoniazid and rifampicin; XDR-TB is defined as MDR-TB, and resistance to any fluoroquinolone, and to any of the three second-line injectables (amikacin, capreomycin, and kanamycin).

S2 Table

Phenotypic drug susceptibility tests status by lineage

Lineage	N	%	Susceptible	Drug-resistant	MDR-TB	XDR-TB
1	1851	11.1	1492	203	150	6
2	3653	21.9	1445	479	1572	157
3	2830	17.0	2162	215	425	28
4	8354	50.1	5665	935	1601	153
Overall	16688	100.0	10764	1832	3748	344
			64.5%	11.0%	22.5%	2.1%

Drug-resistant refers to non-MDR-TB/-XDR-TB resistance; MDR-TB is defined as resistance to isoniazid and rifampicin; XDR-TB is defined as MDR-TB, and resistance to any fluoroquinolone, and to any of the three second-line injectables (amikacin, capreomycin, and kanamycin).

S3 Table

Phenotypic drug susceptibility testing results

Drug	No. tests	% of 16,688	Resistant	%
Rifampicin	16507	98.9	4462	27.0
Isoniazid	16422	98.4	5215	31.8
Ethambutol	14830	88.9	2576	17.4
Pyrazinamide	11968	71.7	1813	15.1
Streptomycin	5213	31.2	1338	25.7
Ofloxacin	1993	11.9	506	25.4
Kanamycin	1843	11.0	639	34.7
Capreomycin	1731	10.4	389	22.5
Amikacin	1435	8.6	335	23.3
Ethionamide	940	5.6	329	35.0
Moxifloxacin	885	5.3	104	11.8
PAS	407	2.4	43	10.6
Ciprofloxacin	400	2.4	63	15.8
Cycloserine	391	2.3	105	26.9

PAS = para-aminosalisylic acid

S4 Table

Predictive accuracy and Area under the ROC Curve (AUC) for models (maximum value per prediction measure is bolded)

Drug	LR-KDG			CT-KDG			CT-ALL			GBT-ALL			GBT-CRM		
	**NPV**	**PPV**	**AUC**	**NPV**	**PPV**	**AUC**	**NPV**	**PPV**	**AUC**	**NPV**	**PPV**	**AUC**	**NPV**	**PPV**	**AUC**
INH	94.2	**97.9**	93.7	94.2	**97.9**	93.4	94.2	**97.9**	93.4	94.5	97.7	95.8	**95.8**	97.4	**96.7**
RIF	94.1	**98.7**	91.2	94.1	**98.7**	91.2	94.1	**98.7**	91.2	94.1	**98.7**	95.3	**96.0**	96.8	**97.9**
PZA	86.7	**100.0**	60.7	86.7	**100.0**	60.8	88.6	82.8	73.7	89.8	91.8	87.0	**94.2**	78.0	**95.5**
EMB	**96.5**	72.9	89.9	95.7	74.5	87.4	95.7	74.5	87.4	95.9	**77.2**	94.0	96.1	75.6	**95.8**
STM	90.9	**91.8**	87.3	91.0	87.7	87.1	90.8	90.0	88.4	91.1	90.2	92.2	**93.3**	87.3	**94.0**
AMK	**94.4**	**98.1**	91.1	**94.4**	**98.1**	90.0	**94.4**	**98.1**	90.0	**94.4**	**98.1**	94.5	**94.4**	**98.1**	**96.4**
CAP	91.4	82.0	84.0	91.4	82.0	82.5	91.4	82.0	82.5	92.1	83.8	90.2	**92.8**	**85.5**	**93.4**
KAN	86.3	**98.1**	88.5	86.3	**98.1**	86.7	89.9	95.8	90.4	89.2	95.7	92.9	**90.0**	96.6	**96.8**
CIP	**98.5**	**92.8**	95.6	**98.5**	**92.8**	95.6	**98.5**	**92.8**	95.6	97.0	92.3	92.9	97.0	92.3	**99.7**
OFL	94.0	91.5	88.8	94.0	91.5	88.8	94.0	91.5	88.8	**94.3**	**91.6**	92.0	94.2	89.5	**93.3**
MOX	**96.7**	47.6	79.9	**96.7**	47.6	79.9	95.2	**70.0**	93.0	95.7	57.1	**97.4**	95.7	66.6	97.2
ETH	**85.3**	62.5	79.7	**85.3**	62.5	75.6	85.2	66.2	80.4	83.8	83.0	85.4	84.5	**84.9**	**88.4**
CYS*	-	-	-	-	-	-	73.5	72.7	69.8	77.8	75.0	80.6	**79.0**	**76.5**	**83.8**
PAS**	87.8	-	50.0	**90.0**	**100.0**	60.0	87.8	-	67.9	88.9	**100.0**	**82.6**	**90.0**	**100.0**	82.5
MDR	96.0	88.9	96.5	96.0	88.9	91.4	96.0	88.9	91.4	96.0	**91.0**	97.1	**97.2**	89.5	**97.4**

* No known drug resistance SNPs for CYS were included in the KDG models; CT-KDG is a classification tree (CT) fitted to a dataset with SNPs that are known to be associated with drug resistance (derived from (24)); LR-KDG is a Logistic Regression model applied to the same SNP set as CT-KDG; CT-ALL and GBT-ALL are respectively a CT and Gradient Boosted Tree (GBT) applied to a dataset that includes all genome-wide SNPs, except those linked to resistance for other drugs ("co-occurrent resistance markers"); GBT-CRM is a GBT that is applied to all genome-wide SNPs; PPV=Positive Predicted Value, NPV=Negative Predicted Value, AUC=Area under the ROC Curve; ** PPV for PAS for LR-KDG and CT-ALL could not be calculated as sensitivity was 0. Reported outcomes are the performance on the test-set; RIF=rifampicin, INH=isoniazid, EMB=ethambutol, PZA=pyrazinamide, CIP=ciprofloxacin,OFL= ofloxacin, MOX=moxifloxacin, AMK=amikacin, KAN=kanamycin, CAP=capreomycin, PAS=para-aminosalisylic acid (PAS), CYS=cycloserine, ETH=ethionamide; MDR is multi-drug resistant TB.

S5 Table

Summary of drugs and mutations in *TBProfiler* library* used in this study

Drug	Locus	Gene	SNPs	Indels
Rifampicin	Rv0667	*rpoB*	94	25
	Rv0668	*rpoC*	8	-
Isoniazid	*Rv1483*	*fabG1*	11	-
	Rv1484	*inhA*	13	-
	Rv1908c	*katG*	226	37
	Rv2245	*kasA*	4	-
	Rv2428	*ahpC*	21	-
Ethambutol	*Rv1267c*	*embR*	20	-
	Rv3793	*embC*	25	-
	Rv3794	*embA*	9	6
	Rv3795	*embB*	127	1
Pyrazinamide	*Rv1630*	*rpsA*	3	-
	Rv2043c	*pncA*	280	87
	Rv3601c	*panD*	10	1
Streptomycin	*Rv0682*	*rpsL*	16	-
	Rv3919c	*gid*	2	26
	rrs	*rrs*	19	-
Ethionamide	*Rv1483*	*fabG1*	3	-
	Rv1484	*inhA*	3	-
	Rv3854c	*ethA*	33	42
	Rv3855	*ethR*	2	-
Amikacin	*rrs*	*rrs*	6	-
Capreomycin	*Rv1694*	*tlyA*	16	13
	rrs	*rrs*	4	-
Kanamycin	*Rv2416c*	*eis*	10	-
	rrs	*rrs*	4	-
FQ	*Rv0005*	*gyrB*	26	-
	Rv0006	*gyrA*	21	-
PAS	*Rv2447c*	*folC*	18	-
	Rv2671	*ribD*	1	-
	Rv2754c	*thyX*	1	-
	Rv2764c	*thyA*	19	5
Cycloserine	*Rv2780*	*ald*	-	12
	Rv3423c	*alr*	3	-

* https://github.com/jodyphelan/tbdb;; indels = insertions and deletions, FQ = Fluoroquinolones, PAS = Para-aminosalicylic acid

S6 Table

Comparison between Gradient Boosted Tree model (GBT-CRM), TB-Panel, TB-Profiler and GWAS study

Drug	TB-Panel*			TB-Profiler**			GWAS***			GBT-CRM****		
	Sens	Spec	Acc.	Sens	Spec	Acc.	Sens	Spec	Acc.	Sens	Spec	Acc.
INH	88.0	97.0	94.1	93.7	98.1	96.7	92.2	98.6	96.6	91.1	98.8	96.3
RIF	84.1	99.4	95.3	95.9	98.2	97.6	92.9	98.6	97.1	88.8	98.9	96.2
PZA	19.9	98.8	86.4	87.6	96.7	95.3	39.4	98.2	89.0	69.7	96.1	91.8
EMB	84.1	93.3	91.7	92.1	91.7	91.8	89.0	92.9	92.2	82.8	94.2	92.1
STM	81.4	81.5	81.5	78.0	96.3	91.6	70.2	98.1	90.9	79.8	96.0	91.9
AMK	82.3	86.5	85.5	86.0	98.3	95.4	86.0	98.6	95.7	80.5	99.5	95.1
CAP	76.3	84.9	83	84.7	95.9	93.4	78.7	96.7	92.6	74.6	96.2	91.3
KAN	84.9	86.9	86.2	92.0	96.8	95.1	86.2	98.2	94.0	82.2	98.2	92.1
CIP	80.9	98.2	95.5	90.6	98.0	96.8	84.1	98.8	96.5	85.7	98.5	96.2
OFL	81.0	97.4	93.2	90.1	96.5	94.9	83.8	97.6	94.1	81.0	97.0	93.2
MOX	81.7	92.7	91.4	86.0	91.9	91.2	81.7	93.6	92.2	53.3	97.5	93.7
ETH	76.5	75.4	75.8	89.5	67.4	75.1	55.7	86.6	75.7	68.1	93.4	84.6
CYS	-	-	-	43.0	92.5	79.2	33.3	98.3	80.8	50.0	92.4	78.4
PAS	9.3	97.8	88.4	23.8	96.7	89.0	48.8	95.3	90.4	20.0	100.0	90.2
MDR	79.7	97.7	93.8	94.1	98.3	97.3	89.8	98.7	96.5	90.4	96.9	95.5

GBT-CRM is a Gradient Boosted Tree (GBT) that is applied to all genome-wide SNPs (including co-occurrent resistance markers); * List of TB Profiler panel mutations with minor allele frequency > 0.5% in the dataset, and applied on a "rule-in" basis; ** TB-Profiler prediction (24); *** GWAS approach as described in (12) but re-run on the 17k dataset used in this study; ****Reported outcomes for GBT-CRM is based on the performance when applied to the test-set; RIF=rifampicin, INH=isoniazid, EMB=ethambutol, PZA=pyrazinamide, CIP=ciprofloxacin,OFL= ofloxacin, MOX=moxifloxacin, AMK=amikacin, KAN=kanamycin, CAP=capreomycin, PAS=para-aminosalisylic acid (PAS), CYS=cycloserine, ETH=ethionamide, Sens=Sensitivity, Spec=Specificity, Acc=Accuracy, MDR = multi-drug resistant TB.

S7 Table

Comparison between Gradient Boosted Tree model (GBT-CRM) and average scores across other machine learning studies*

	Sens GBT-CRM	Spec GBT-CRM	AUC GBT-CRM	Sens Other (avg.)	Spec Other (avg.)	AUC - Other (avg.)	Difference (Sens)	Difference (Spec)	Difference (AUC)
INH	91.1	98.8	96.7	93.4	96.1	97.6	-2.3	2.7	-0.9
RIF	88.8	98.9	97.9	94.7	97.0	98.4	-5.9	1.9	-0.5
PZA	69.7	96.1	95.5	83.7	90.3	92.9	-14.0	5.8	2.6
EMB	82.8	94.2	95.8	93.5	93.8	97.4	-10.7	0.4	-1.6
STM	79.8	96.0	94.0	88.2	91.3	93.5	-8.4	4.8	0.5
AMK	80.5	99.5	96.4	83.4	90.3	93.2	-2.9	9.2	3.2
CAP	74.6	96.2	93.4	68.2	89.2	83.1	6.4	7.0	10.3
KAN	82.2	98.2	96.8	80.8	91.5	90.2	1.4	6.7	6.6
CIP	85.7	98.5	99.7	87.9	91.7	93.8	-2.2	6.8	5.9
OFL	81.0	97.0	93.3	81.6	92.2	91.3	-0.6	4.8	2.0
MOX	53.3	97.5	97.2	87.3	90.4	91.8	-34.0	7.1	5.4
ETH	68.1	93.4	88.4	90.6	85.6	92.2	-22.5	7.8	-3.8
MDR	90.4	96.9	97.4	96.2	96.5	99.4	-5.8	0.4	-1.9

GBT-CRM is a Gradient Boosted Tree (GBT) that is applied to all genome-wide SNPs (including co-occurrent resistance markers); * other studies with results found in references (22,23,26,27). For study (27) the performance is the DeepAMR model. Note: Not all studies included all drugs; Reported outcomes for the GBT-CRM is the performance based on the application to the test-set ; RIF=rifampicin, INH=isoniazid, EMB=ethambutol, PZA=pyrazinamide, CIP=ciprofloxacin,OFL= ofloxacin, MOX=moxifloxacin, AMK=amikacin, KAN=kanamycin, CAP=capreomycin, PAS=para-aminosalisylic acid (PAS), CYS=cycloserine, ETH=ethionamide, Sens = Sensitivity, Spec = Specificity, AUC=Area under the ROC Curve

S8 Table

Comparison between Gradient Boosted Tree model (GBT-CRM) and maximum scores in other machine learning studies*

	Sens GBT-CRM	Spec GBT-CRM	AUC GBT-CRM	Sens Other (max)	Spec Other (max)	AUC Other (max)	Difference (Sens)	Difference (Spec)	Difference (AUC)
INH	91.1	98.8	96.7	97.0	98.4	99.0	-5.9	0.4	-2.3
RIF	88.8	98.9	97.9	97.0	97.8	99.0	-8.2	1.1	-1.1
PZA	69.7	96.1	95.5	88.1	91.2	95.0	-18.4	4.9	0.5
EMB	82.8	94.2	95.8	97.0	96.0	99.0	-14.2	-1.8	-3.2
STM	79.8	96.0	94.0	90.1	94.2	95.2	-10.3	1.8	-1.2
AMK	80.5	99.5	96.4	89.5	90.8	95.0	-9.0	8.7	1.4
CAP	74.6	96.2	93.4	71.9	92.7	85.5	2.7	3.5	7.9
KAN	82.2	98.2	96.8	81.1	93.5	92.5	1.1	4.7	4.3
CIP	85.7	98.5	99.7	96.0	98.0	98.0	-10.3	0.5	1.7
OFL	81.0	97.0	93.3	96.0	93.7	95.0	-15.0	3.3	-1.7
MOX	53.3	97.5	97.2	95.0	93.0	95.0	-41.7	4.5	2.2
ETH	68.1	93.4	88.4	90.6	85.6	92.2	-22.5	7.8	-3.8
MDR	90.4	96.9	97.4	96.3	98.0	100	-5.9	-1.1	-2.6

GBT-CRM is a Gradient Boosted Tree (GBT) that is applied to all genome-wide SNPs (including co-occurrent resistance markers); * other studies with results found in references (22,23,26,27). For study (27) the performance is the DeepAMR model. Note: Not all studies included all drugs; Reported outcomes for the GBT-CRM is the performance based on the application to the test-set ; RIF=rifampicin, INH=isoniazid, EMB=ethambutol, PZA=pyrazinamide, CIP=ciprofloxacin,OFL= ofloxacin, MOX=moxifloxacin, AMK=amikacin, KAN=kanamycin, CAP=capreomycin, PAS=para-aminosalisylic acid (PAS), CYS=cycloserine, ETH=ethionamide, Sens = Sensitivity, Spec = Specificity, AUC=Area under the ROC Curve

S9 Table

The machine learning parameter settings

Classifier	Parameters
Decision Tree Classifiers (“CT”)	Function to measure the quality of a split = Gini,<br>Minimum of samples required before making splits=3,<br>Minimum of samples required for leaf nodes=3,<br>The minimum weighted fraction of the sum total of weights (of all the input samples) required to be at a leaf nod =0,<br>The number of covariates to consider when looking for the best split=None,<br>max_leaf_nodes=None,<br>Minimum impurity decrease required for splits=0.0,<br>Minimum impurity threshold=0,<br>Class weighting=None.
Gradient Boosted Tree Classifier (“GBT”)	Boosting learning rate=0.1<br>Booster='gbtree'<br>Min. loss reduction required for further partition on a leaf node =0<br>Minimum sum of instance weight(hessian) needed in a child =1<br>Maximum delta step we allow each tree’s weight estimation to be=0<br>Subsample ratio of columns when constructing each tree=1<br>Subsample ratio of columns for each split, in each level=1<br>L1 regularization term on weights=0<br>L2 regularization term on weights=1<br>Global bias=0.5.
Logistic Regression (“LR”)	Penalty=”L1”<br>Tolerance=0.0001<br>Maximum iterations=100

* see Methods for those parameters that were chosen by cross-validation

S1 Figure

A two-dimensional mutation ranking across drugs created from the outputs of the gradient boosted tree (GBT) models, using the proportion of GBT trees within the overall ensemble they appear in and the information gain associated with their presence. The orange points refer to previously known SNPs (TB-profiler), with the dotted green box as a suggested detection threshold determined by optimizing the discrimination between previously known SNPs and other SNPs across drugs.

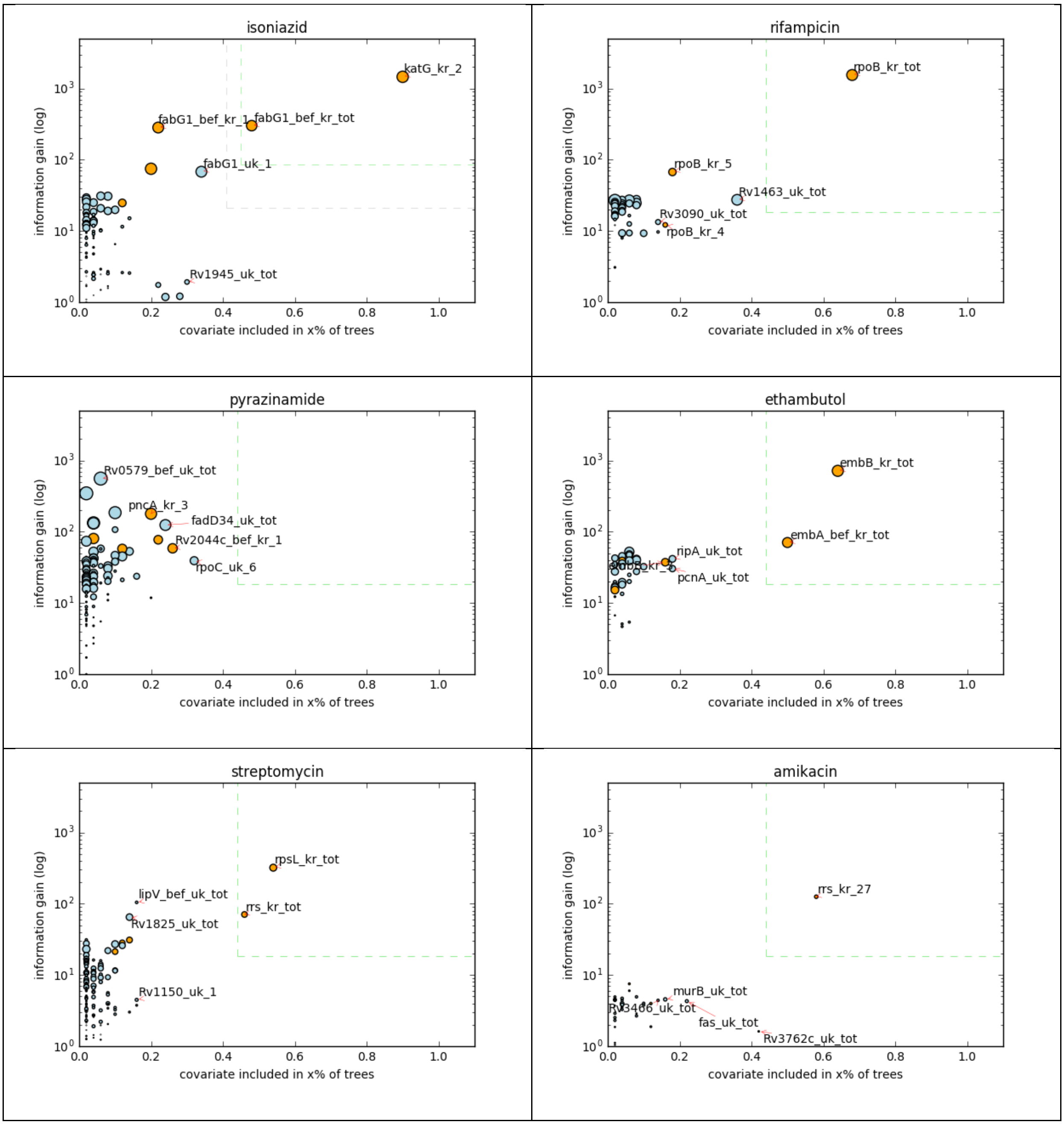

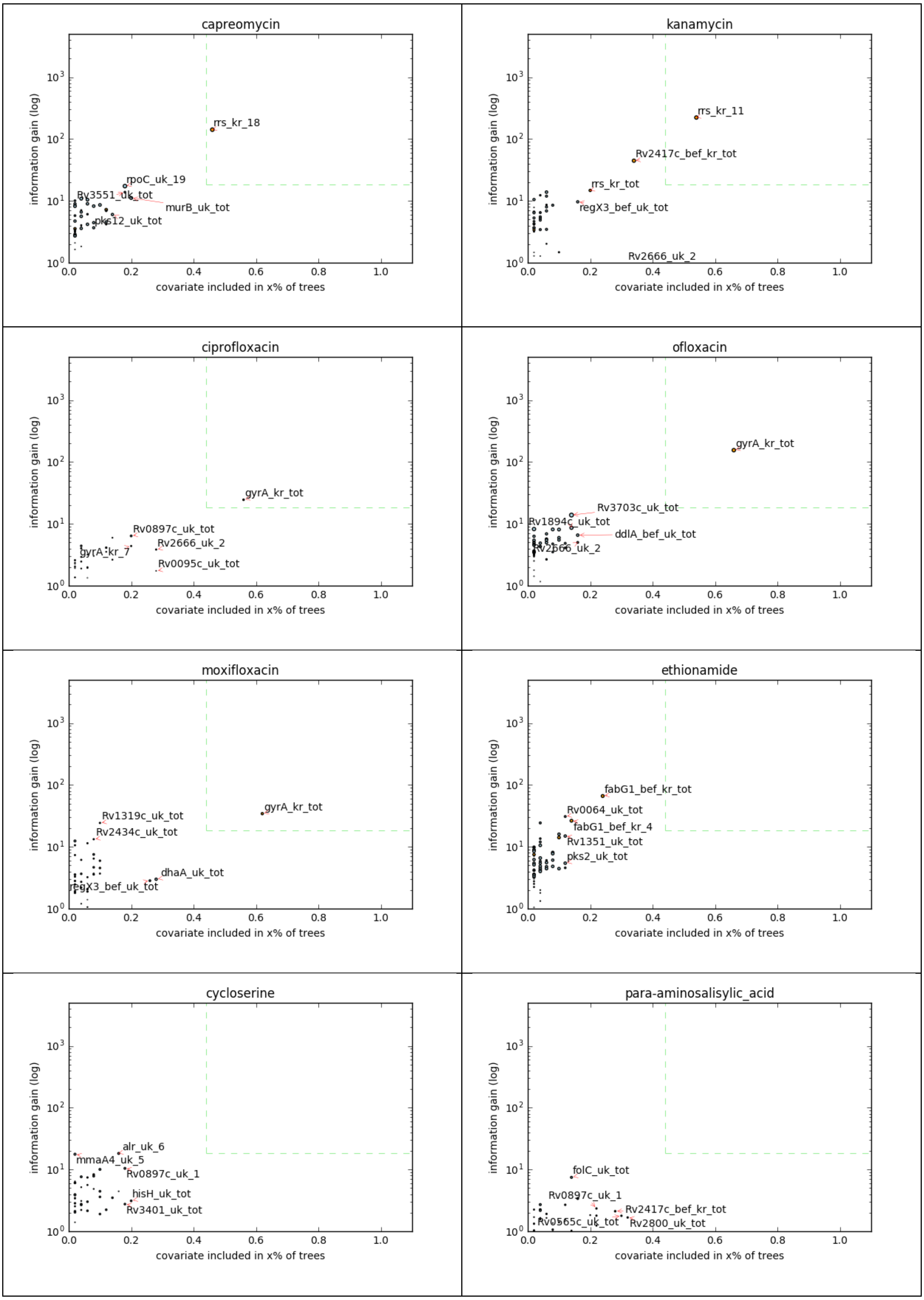
capreomycin
rrs_kr_18
rpoC_uk_19
Rv3551_uk_tot
murB_uk_tot
pks12_uk_tot
kanamycin
rrs_kr_11
Rv2417c_bef_kr_tot
rrs_kr_tot
regX3_bef_uk_tot
Rv2666_uk_2
ciprofloxacin
gyrA_kr_tot
Rv0897c_uk_tot
Rv2666_uk_2
gyrA_kr_7
Rv0095c_uk_tot
ofloxacin
gyrA_kr_tot
Rv3703c_uk_tot
Rv1894c_uk_tot
ddlA_bef_uk_tot
Rv2666_uk_2
moxifloxacin
gyrA_kr_tot
Rv1319c_uk_tot
Rv2434c_uk_tot
dhaA_uk_tot
regX3_bef_uk_tot
ethionamide
fabG1_bef_kr_tot
Rv0064_uk_tot
fabG1_bef_kr_4
Rv1351_uk_tot
pks2_uk_tot
cycloserine
alr_uk_6
mmaA4_uk_5
Rv0897c_uk_1
hisH_uk_tot
Rv3401_uk_tot
para-aminosalisylic_acid
folC_uk_tot
Rv0897c_uk_1
Rv2417c_bef_kr_tot
Rv0565c_uk_tot
Rv2800_uk_tot
information gain (log)
covariate included in x% of trees
